# Utilizing timed categorical recall (naming US cities) for rapid bedside dementia screening

**DOI:** 10.1097/MD.0000000000029518

**Published:** 2022-08-05

**Authors:** Charles R. Joseph, Michael P. Cargill, Chansoon D. Lee

**Affiliations:** a Liberty University College of Osteopathic Medicine, Lynchburg, VA; b Liberty College of Osteopathic Medicine, Lynchburg, VA.

**Keywords:** cognitive testing, dementia, Folstein MMSE, rapid dementia screen, timed categorical recall

## Abstract

The availability of fast validated screening for dementia is a critical clinical need to improve neurologic examination time efficiency. This study validated a 1-minute timed categorical recall (TCR) method, naming as many US cities as possible and compared TCR to the Folstein Minimental Status Exam (MMSE) as a preliminary cognitive screening tool.

Random uncompensated 349 volunteers were recruited ages 18 to 97 from local free clinics, retirement homes, university faculty, and students in Lynchburg, Virginia 2015 to 2020. Participants’ demographic and medical information were collected. After 1 minute preparation, participants were rapidly named as many US cities as possible until they were told to stop (1 minute). The time limitation was withheld in advance. Number of cities and organizational strategies were recorded. Folstein MMSE administration immediately after TCR was administered to 122 subjects recruited in the final 2 study years as a comparison benchmark. A multiple linear regression model and a regression tree model were used to identify important variables for the number of cities named and determine subgroups and their thresholds.

TCR resulted in accuracy rate (0.80), sensitivity (0.78), and specificity (0.81). The global TCR threshold (9 cities named) is superseded by 4 subgroup thresholds, categorized by statistically important variables (age, education level, and number of states visited) as follows:

For those visiting ≥8 states and

1. 18 to 71 ages with a master’s degree or above, the threshold was naming 20 cities;

2. 18 to 29 ages with a bachelor’s degree or below, the threshold was naming 17 cities;

3. 30 to 71 ages with a bachelor’s degree or below, the threshold was naming 10 cities.

For those visiting <8 states *or* for ages 72 to 97 (regardless of education levels and number of states visited), the threshold was naming 8 cities.

American cities are common knowledge across ages and backgrounds, making it a useful bedside screen for dementia. In clinical practice, patients who report fewer cities than the threshold of 9 cities should receive further cognitive testing. If the patient meets the criteria for a subgroup, then the higher subgroup thresholds apply. TCR is a more time-efficient preliminary dementia screening tool with improved sensitivity and similar specificity compared with MMSE.

## 1. Introduction

Screening for mild cognitive impairment or dementia while performing a complete neurologic examination decreases efficiency in most clinical settings.^[1]^ Although more common in advanced age groups dementia in young is not uncommon, resulting from head injury or disease. There are several methods of validated formal screening that are in use and have been compared in recent reviews but are too time consuming for preliminary rapid screening as part of a general clinical examination.^[2,3]^ Administration of a timed categorical recall test (TCR) can mitigate the aforementioned problem. TCR evaluates not only information in long-term storage within the inferolateral temporal lobe (TL), but also prefrontal working memory, organizational processes, item selection skills, speech pathways, and the accelerator (rapidity of answering) function.^[4]^ Anatomically, the dorsolateral prefrontal cortex (DLPFC), ventrolateral prefrontal cortex (VLPFC), TL, and frontal-temporal-parietal speech and thalamic accelerator pathways are assessed.^[5–9]^ The VLPFC has been found through functional magnetic resonance imaging (MRI) testing to be associated with item selection in working memory, while the DLPFC is associated with organizational processing.^[4]^ Both areas must function to provide successful and efficient long-term storage and recall. The element of psychological pressure to name the maximum possible number of cities as quickly as possible provides a useful assessment of the attentional accelerator system within the basal ganglia and thalamic structures.^[4,6,7]^

Our primary goal is to validate this TCR (naming US cities) as a rapid bedside dementia screening method across a broad range of adult ages. The present study assessed these potential independent variables on performance: advanced age (e.g., >70 years old), gender, education level, number of states visited, using an organized recall method (e.g., geographic by region or city size), head injury history, cancer history, taking psychotropic medications, or alcohol use.

## 2. Methods

The present study proposed and validated a TCR for a broad age range (18–97), in which subjects are asked to name US cities as rapidly as possible. It requires minimal basic patient instruction by the examiner and only 2 minutes total (1 minute to collect their thoughts and 1 minute to respond) to perform. The examiner should not divulge the response time limit in advance to the patient to reduce the potential for test anxiety. Howard et al validated a timed recall test involving the naming of animals in a college student cohort sample.^[10–12]^ They studied written categorical recall tests of multiple-category associations but did not find differences among three broad age ranges, 18 to 39, 40 to 59, and 60 to 79.^[13]^ However, multiple-category recall requires a larger fund of knowledge and can introduce bias based on background experience. Written responses also add another potential confounder related to spelling and writing abilities. Therefore, we tested one item category and eliminated written responses to avoid them. The study validated TCR in terms of accuracy, sensitivity, and specificity, and determined thresholds for all subjects as well as subgroups based on the significant variables. In addition, the correlation between TCR and the Folstein Minimental Status Exam (MMSE) score was examined.

### 2.1. Participants

Uncompensated volunteers were recruited between the ages of 18 and 97 from a variety of venues, including a local free clinic (staff and patients’ family members), medical outreach events (patients and family), local retirement homes (residents), and university students and faculty volunteers from 2015–2016 to 2018–2020. In 2015 to 2016, 227 participants between the ages of 18 and 81 participated in the study, and in 2018 to 2020, 122 patients from ages 40 to 97 participated. The total sample size for this study was 349 participants. The R package *pwr* was used with the significance level = 0.05 and power = 0.90 for a multiple regression model.^[14]^ The determined sample size was 33, and the obtained sample size (349) was enough to achieve the power. This predictive study was conducted for quantifying the number of cities named by subjects, classified using identified important independent variables.

### 2.2. Procedures

IRB approval was obtained (LU IRB 2207.051515 and CHIRB0419). Informed verbal consent was given before the testing. Afterward, brief questions were asked about demographics, travel experience, and health-related questions. The questions included age, gender, education level, number of states visited, years lived in the United States, history of head injury, stroke, intracranial tumor, cancer history, medications taken currently, brain irradiation, chemotherapy, and alcohol use (see Appendix, http://links.lww.com/MD/H175). Each participant’s testing was conducted in a private setting by medical student co-investigators, and responses were recorded without identifiers. After completing the initial questionnaire, the subjects were asked to name as many US cities as quickly as possible until told to stop. All were given 1 minute to collect their thoughts before they were told to begin. The timed city recall was 1 minute in duration; the subjects were not informed of this in advance. The standard question asked was, “I will ask you to name as many US cities (not states) as quickly as possible until I tell you to stop. Now please take 1 minute to collect your thoughts and I will tell you when to begin.” The investigator tallied the number of responses and identified any organization strategy used by the subject (i.e., geographic by region or city size vs random recall). The group studied in 2018 to 2020 was given the Folstein MMSE immediately after taking the TCR test. All data were transferred to a spreadsheet for statistical analysis.

### 2.3. Statistical analysis

A multiple linear regression model was used to investigate which variables were statistically significant for TCR. For the regression model, the number of cities named was the dependent variable, and 9 out of 12 independent variables (listed above) were used. Two variables (brain irradiation and chemotherapy) were excluded from the data due to missing values. Also, the variable of years lived in the United States was excluded for the regression model because it had a strong positive correlation (*r* = 0.8) with the variable of the number of states visited, which can lead to multicollinearity that suppresses the impact one of those variables would otherwise have on the dependent variable.

Additionally, a regression tree model was used to determine criteria and thresholds for subgroups. For this, the classification and regression tree (CART) was selected because it is a critical ground for other tree algorithms and is still widely used.^[15]^ The Pearson correlation coefficient was also utilized to examine a strongly positive association between the Folstein MMSE and TCR, which is a convergent validity showing that both tests measure cognitive function.

This study employed three evaluation measures to validate the TCR approach: accuracy rate, sensitivity, and specificity. The accuracy rate is the proportion of correctly classified cases: a case diagnosed by TCR corresponds to MMSE’s result. Sensitivity is the ability of the TCR to correctly identify patients with cognitive impairment based on the MMSE. Specificity is the ability of the TCR to correctly identify patients without cognitive impairment with regard to the MMSE. For validation, we chose threshold of 24 for MMSE.^[16]^ Data analysis was conducted using R and the *rpart* package for the CART tree model.^[17,18]^

## 3. Results

### 3.1. Descriptive statistics

**Figure 1.**
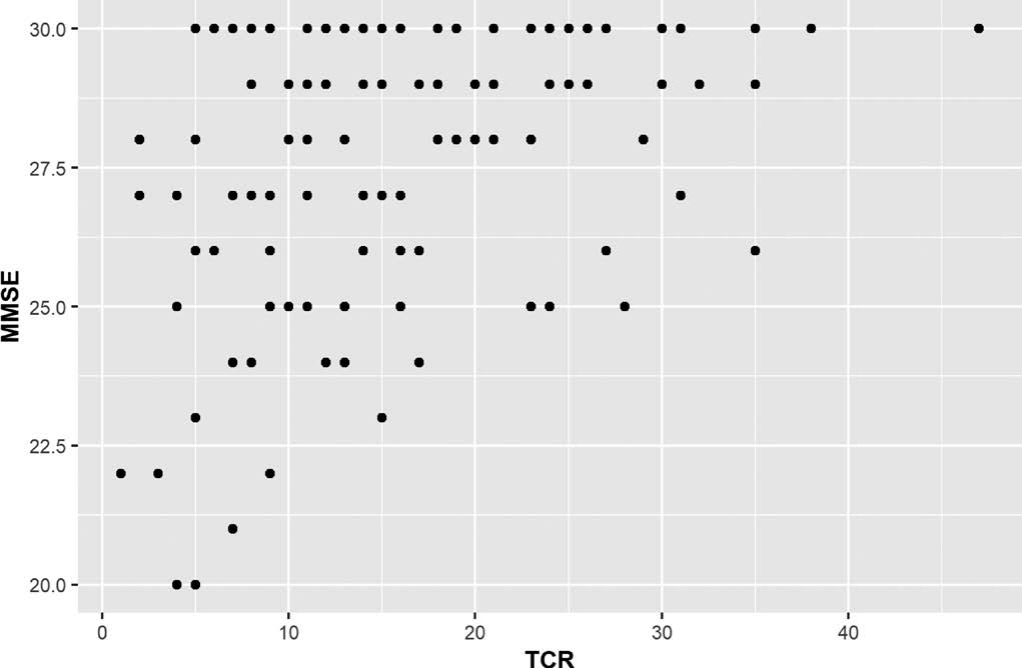
Scatterplot showing the correlation between timed categorical recall (TCR) and Minimental status exam (MMSE).

Table 1 shows the participants’ characteristics and descriptive statistics for variables used in the study. The mean number of cities named by TCR participants was 18 with a relatively large standard deviation (SD), 9.2. The mean of participants’ Folstein MMSE scores, which were measured only in the 2018 to 2020 data collection (N = 122), was 27 with an SD of 2.5. Using the same data, a positive correlation between TCR and MMSE (Fig. 1) was statistically significant, having a Pearson correlation coefficient *r* (120) = 0.41, *P* < .001, and 95% CI [0.25, 0.55]. In terms of convergent validity, TCR correlated significantly and positively with MMSE. The average age of all participants was 47 years old, and more females participated in the testing (57.6%). The majority of the participants (75.4%) completed at least 12th grade, and 61.6% of the participants used organized strategies (i.e., geographic by region and city size) in the TCR process.

**Table 1:** Participants’ descriptive statistics.

Variable		M (SD)
TCR		18.573 (9.177)
Folstein MMSE*		27.680 (2.504)
Age in years		47.461 (18.724)
Number of states visited		15.169 (13.108)
**variable**	**Category**	**n (%)**
Gender	Male	148 (42.4%)
	Female	201 (57.6%)
Education	Under 12th grade	28 (8.0%)
	12th grade	123 (35.3%)
	Undergraduate	140 (40.1%)
	Masters	42 (12.0%)
	Doctoral	16 (4.6%)
Head Injury (stroke, tumor)	Yes	85 (24.4%)
	No	264 (75.6%)
Cancer	Yes	37 (10.6%)
	No	312 (89.4%)
Medication	None	173 (49.6%)
	HTN, cholesterol, cardiac, thyroid	135 (38.7%)
	Opiates or psychiatric medications	41 (11.7%)
ETOH use	None	205 (58.8%)
	1–6 standard drinks/wk	109 (31.2%)
	7 or more standard drinks/wk	35 (10.0%)
Naming strategy	Geographical (city/size)	215 (61.6%)
	Random	134 (38.4%)

### 3.2. Important variables and a global threshold for TCR

Out of 9 variables, those statistically significant for TCR were age, education level, number of states visited, and use of organized strategy (see Table 2). About 36% of the variation in the number of cities named in TCR was accounted for by the regression model of the nine variables. A threshold for TCR, nine cities named, was obtained by using one SD below the mean, which is a minimally accepted cutoff method in clinical settings.^[19]^ The threshold of 9 is called a global threshold for TCR in this study to differentiate the global threshold from subgroup thresholds, which are described in the next section. Using the global threshold of 9, TCR resulted in an accuracy rate of 0.80, a sensitivity of 0.78, and a specificity of 0.81.

**Table 2:** Regression of variables on number of cities named in timed categorical recall (TCR).

Variable	B	SE	*t*	*P*
Age	-0.120	0.027	-4.501	.000***
Gender (male)	1.409	0.869	1.621	.106
Education (12th grade)	3.095	1.571	1.970	.050*
Education (bachelors)	5.308	1.663	3.192	.002**
Education (masters)	9.507	1.885	5.042	.000***
Education (doctorate)	11.374	2.459	4.626	.000***
Number of states visited	0.193	0.034	5.666	.000***
Head injury (yes)	-0.015	0.956	-0.016	.988
Cancer (yes)	0.074	1.376	0.053	.957
Medication (HTN, cholesterol)	-0.761	0.950	-0.802	.423
Medication (opiates)	-0.916	1.391	-0.659	.511
ETOH (1–6 times)	1.478	0.927	1.595	.112
ETOH (7 or more)	0.323	1.420	0.227	.820
Organized strategy (geographic/city size)	4.527	0.822	5.508	.000***
*R* ^2^	0.386			
?*R*^2^	0.360			

### 3.3. Subgroup thresholds for TCR

**Figure 2.**
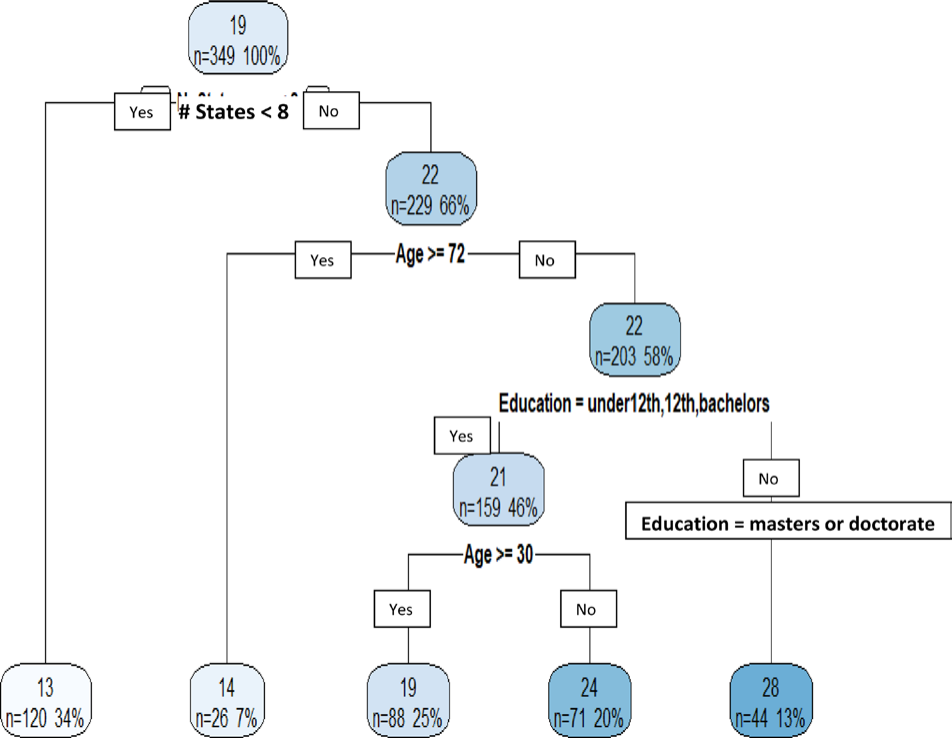
Regression tree model for number of cities named in timed categorical recall (TCR). . Each node (rounded square) in the tree model has the predicted number of cities named in timed categorical recall (TCR), the number of subjects (n) belonging to the node, and percent of the sample. An interpretation of the tree is that if a patient visited less than 8 states, then the patient’s predicted number of cities named is 13 regardless of his/her age and education level. Thirty-four percent of the participants (120 subjects) in the sample responded they visited <8 states. If a patient visited ≥ 8 states and his or her age is younger than 72 years old with masters’ or doctorate degree, then the patient’s predicted number of cities named is 28.

Figure 2 shows that the CART tree model also selected the number of states visited, age, and education as the important variables, whereas the variable of the organized naming strategy was not. Compared to the multiple linear regression model, the regression tree model is more effective in identifying important variables, obtaining the important variables’ thresholds, and predicting values for the dependent variable, which in this study is the number of cities named in TCR. This study employed important variables and their thresholds from the tree model to form subgroups for TCR. According to the tree model in Figure 2, subjects were first divided into two groups, subjects who visited ≥8 states and subjects who visited <8 states. Those visiting ≥8 states were divided into nine subgroups based on three age groups and three education levels. The variables of age and education were categorized into three levels: age into [18–29], [30–71], and [72–97]; formal education into low (12th grade or less), medium (bachelor’s degree), and high (masters and doctoral degrees). As shown in Table 3, the total categorization resulted in 10 subgroups, which can be condensed into 4 subgroups based on shared thresholds. Thresholds for Groups 1 to 4 were determined using one SD below the mean of the number of cities named in TCR. Those visiting <8 states or who were in the 72 to 97 age group were treated as one group, as their performance was identical. The four subgroups and their thresholds are as follows:

**Table 3:** Thresholds for subgroups 1–4.

	Age (n)		
(a) Number of states visited =8			
Education (n)	18–29 (81)	30–71 (122)	72–97 (26)
Low: up to 12th (62)	17 (G2)	10 (G3)	8 (G4)
Medium: bachelors (115)	17 (G2)	10 (G3)	8 (G4)
High: graduates (52)	20 (G1)	20 (G1)	8 (G4)
	**All age groups (n = 120)**		
(b) Number of states visited			
All education levels	8 (G4)		

1. *Group 1*: (# of states visited ≥8) and (age 18–71) and (education = high), threshold was 20 cities named;
2. *Group 2*: (# of states visited ≥8) and (age 18–29) and (education = low or medium), threshold was 17 cities named;
3. *Group 3*: (# of states visited ≥8) and (age 30–71) and (education = low or medium), threshold was 10 cities named;
4. *Group 4*: (# of states visited <8) or (ages 72–97 regardless of education and # of states visited), threshold was 8 cities named.

**Figure 3.**
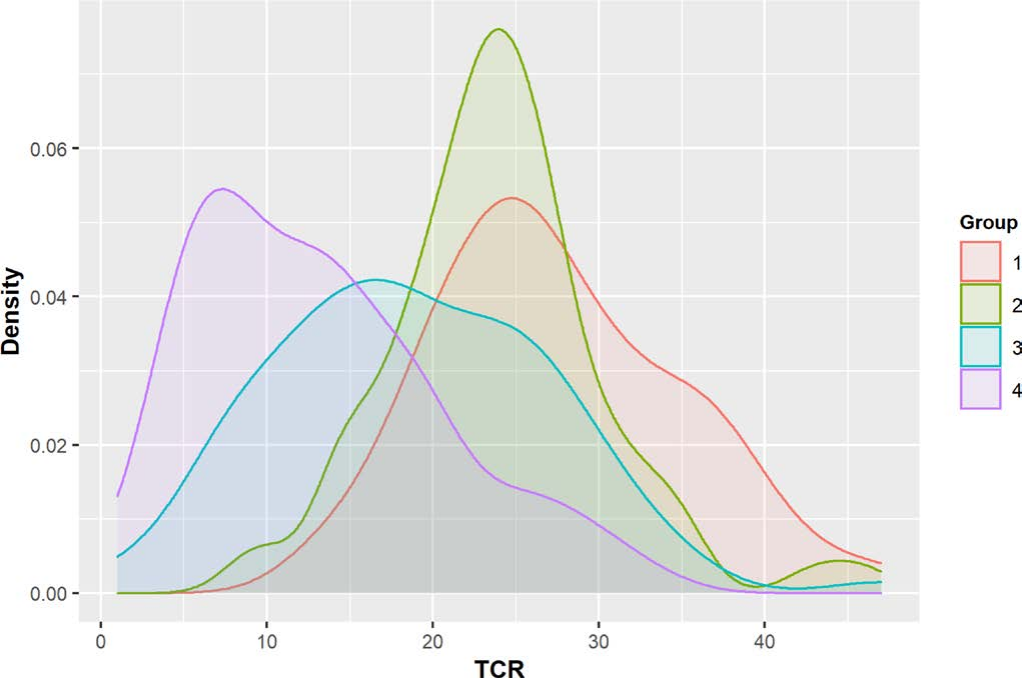
Distributions of timed categorical recall (TCR) for four subgroups. *Note*. Ranges of timed categorical recall (TCR) for subgroups: Group 1 [14, 47]; Group 2 [9, 46]; Group 3 [1, 47]; Group 4 [1, 34].

The TCR thresholds for all subgroups showed the same evaluation measure rates as the global threshold: an accuracy rate of 0.80, a sensitivity of 0.78, and a specificity of 0.81. In Figure 3, density curves show distributions of TCR for the four subgroups. Most of the distributions were positively skewed. The major peak for Group 1 was the highest value of TCR followed by Group 2, 3, and 4. TCR scores in Group 3 were spread out over the entire TCR scores, ranging between 1 and 47.

## 4. Discussion

Time is essential in the clinical practice of neurology and is especially important when evaluating cognitive function. A rapid and sensitive screening test for dementia is a valuable tool for improving examination efficiency. Compared to the MMSE, TCR shows increased sensitivity and comparable specificity in a fraction of the time (2 minutes for TCR vs 15 minutes for MMSE) the latter method requires.^[5]^ This study identified important variables for TCR and determined an absolute threshold of normal responses across all age groups (9 cities named). In addition to the global threshold for TCR, four subgroup thresholds were determined based on the most important independent variables from a decision tree model (Table 3). The study showed that the number of cities named in TCR could be predicted well by age, education levels, and travel experience. Among three variables, travel experience, which was measured by the number of states visited, was the most important predictor for the number of cities named in TCR. The significant predictive performance of higher education level on higher number of cities names in TCR could be explained by superior learned organizational strategies. Both education and travel experience increase the fund of knowledge stored in long-term memory and thus improve performance. The reduced recall with advancing age follows the general pattern of senescence of cognitive function.

As an initial cognitive screen, TCR of cities can easily be incorporated along with testing orientation and short-term memory in far less time than performing the MMSE or Montreal Cognitive Assessment (MoCA). Since the names of cities are common knowledge for the population at large, the recall of them is ideally suited for testing over a broad range of educational and experiential circumstances. This method not only provides information about long-term semantic storage but also assesses attention, working memory, speech pathways, thalamic accelerator function, and the organizational efficiency of retrieval processing.^[20,21]^ TCR examines the above-mentioned parameters quickly. Additionally, the TCR method mitigates performance anxiety by purposely not informing participants of the time limitation of testing, thereby reducing the influence of a deadline distractor. By phrasing the exercise, “I want you to name as many US cities, not states, as fast as you can until I will tell you to stop. Take a minute to collect your thoughts and I will tell you when to begin,” performance anxiety, a potential confounder, is minimized.^[22]^ Interpreting the results includes first determining whether the global threshold of naming cities is met, then referring to the demographic group that applies to the patient (Table 3), the higher threshold that applies supersedes the global threshold. Should the patient not name the number of cities for their respective threshold, then further formal cognitive testing (MoCA) and screening labs for dementia would be the appropriate next step toward diagnosis.

One of the limitations of the study is the sample generalizability. This study collected data from a single community, although the cross section of subject backgrounds was varied. Also, only English-speaking subjects were studied. Additionally, this quick screen does not address visual-spatial, or calculation domains that are addressed in the lengthier MoCA testing.

In summary, adding TCR to testing orientation and short-term memory has advantages for clinicians when cognitive dysfunction is suspected. The advantages include time efficiency, satisfactory accuracy, sensitivity, specificity, and ease of implementation. In addition, TCR reliably queries multiple aspects of the storage and recall processes simultaneously, such as working memory (VLPFC), recall organizational functions (DLPFC), and semantic memory (TL and various locations), as well as thalamic accelerator function.^[23,24]^ Thus, TCR assesses a large swath of cortical geography, including the prefrontal dorsolateral, prefrontal ventrolateral, inferolateral temporal, basal ganglia, and speech pathways.^[25,26]^ The composite evaluation of frontal and TL functions makes this validated test a useful and versatile dementia screening tool.

## 5. Conclusion

The TCR test is a useful rapid preliminary bedside screen of cognitive dysfunction correlating well with MMSE results in normal subjects. Limitations of this study include restricted geographic representation of volunteers, and inclusion of English speakers only. Nonetheless, potentially it could be applied in any language or country of residence. The higher TCR positive rate compared with MMSE may reflect either increased sensitivity as noted with other cognitive tests such as MoCA, or false positives indicating lower specificity in comparison to MMSE. As in any valid screening examination, however, further definitive testing is required to corroborate and further define the specific cognitive dysfunction. Adjustments in number of correct responses (thresholds) for higher education completion, age <72, and more extensive travel experience for responding, have been quantified (Table 3) and supersede the global threshold response of 9 cities named. The four subgroup demographics and thresholds are listed above.

Even in a busy office, this rapid screen will avoid missed early dementia diagnoses that require further evaluation and potential treatment. The only additional background information required includes knowledge of the number of states visited by the patient, along with their age and education level attained. This can be easily determined by the examiner without need of a separate questionnaire. If a patient is unable to reach their respective threshold of cities named, then further cognitive and laboratory testing will be required to confirm and delineate the etiology of the dysfunction. However, responding in the normal range for the demographic group makes impairment highly unlikely and allays the need for additional testing.

## Author contributions

The following former LUCOM medical students were involved in both data acquisition and analysis: Jasmine Y. Jackson, Emilee Kurtz, Marie D. Arnaout, Siobhan Brady, and Dallas McCorkle.

The following former and current medical students were involved in data acquisition: Sahil Rajesh Vagha, David Wayne Carter, Thang Ngoc Vu, Kongmuang Sawitree, Katayoun Kouchakzadeh, Kaitlyn Elizabeth Kuntzman, Olivia Mae Hopper, Michelle Elizabeth Sausner, Christopher Michael Benhatzel, Lauren Mernin McKinney, Abbie Bennett, Angel Stevenson, Stefanie Chis, Jordan Dehli, Farbod Farhang, Michael Allan Goble, Brent Thomas Hager, Henry Miller, Alexandra Nicole Horta, Joshua Collins, Jeffrey Taylor Moss, Polsha Jules, Patrick Henry Roche, Sarah Ibanez, Sindhuja Tatagari, Victor Demetrio, Vivian Van Anh Tran.

Conceptualization: Charles Joseph.

Data curation: Chansoon Lee, Charles Joseph, Michael Cargill.

Formal analysis: Charles Joseph.

Funding acquisition: Charles Joseph.

Investigation: Charles Joseph.

Methodology: Charles Joseph.

Project administration: Charles Joseph.

Supervision: Charles Joseph.

Validation: Chansoon Lee, Charles Joseph.

Visualization: Charles Joseph.

Writing – original draft: Chansoon Lee, Charles Joseph, Michael Cargill.

Writing – review & editing: Chansoon Lee, Charles Joseph, Michael Cargill.

## Supplementary Material



## Footnotes

* *P* < .05.

** *P* < .01.

*** *P* < .001.
