# Supplementary material for: Utilizing timed categorical recall (naming US cities) for rapid bedside dementia screening
Source: Medicine (Baltimore). 2022 Aug 5;101(31):e29518. doi: 10.1097/MD.0000000000029518 (PMC9351842; doi:10.1097/MD.0000000000029518)
Supplement: Supplementary file 1 [file medi-101-e29518-s001.pdf]

Questionnaire 3—Long term memory test

Intake Questionnaire

Patient's age___ Gender____

What is the highest grade ____ or degree ___ obtained?

How many years have you lived in the US?

Have you traveled outside of Virginia?

If so, how many states have you visited?

Do you have a history of head injury, stroke, or tumor?

Cancer History?____________Meds/XRT ____Type/Location_________

List Daily Medications

Last taken?

ETOH?_____ Amount/day______________Drug Use______

Number of Cities named ____________

Organizational presentation

G= geographic _____________

S= city size

R= random
